# Child Maltreatment and Adolescents’ Exposure to Disorder in Daily Activity Spaces

**DOI:** 10.1007/s10964-025-02211-x

**Published:** 2025-06-23

**Authors:** Susan Yoon, Junyeong Yang, Taylor Napier, Jingyi Wang, Kathryn Maguire-Jack, Bethany Boettner, Christopher R. Browning

**Affiliations:** 1 College of Social Work, The Ohio State University, Columbus, OH USA; 2 Department of Social Welfare, College of Social Sciences, Ewha Womans University, Seoul, South Korea; 3 Graduate School of Education, Kyung Hee University, Seoul, South Korea; 4 Division of Social Science, The Hong Kong University of Science and Technology, Hong Kong, China; 5 School of Social Work, University of Michigan, Ann Arbor, MI USA; 6 Institute for Population Research, The Ohio State University, Columbus, OH USA; 7 Department of Sociology, The Ohio State University, Columbus, OH USA

**Keywords:** Child maltreatment, Activity spaces, Neighborhood disorder, GPS data

## Abstract

Minimal research has explored how child maltreatment shapes adolescents’ exposure to risky environments within youth activity spaces (the places youth visit during their daily routine) which likely influences youth well-being. This study examined the extent to which child maltreatment and neighborhood disorder, individually and collectively, are associated with adolescents’ exposure to physical and social disorder in their activity spaces. Participants were 1215 adolescents (47% boys, M_age_ = 14.26, *SD*_*age*_ = 1.87) recruited from a Midwestern city in the United States. Multilevel modeling was conducted. The results indicated a positive association between child maltreatment and disordered activity spaces, with no significant moderation by residential neighborhood-level disorder. The findings point to the need for enhanced maltreatment prevention efforts and targeted interventions to mitigate environmental risks for adolescents with maltreatment histories.

## Introduction

Child maltreatment, including experiences of physical, psychological, and sexual abuse, and neglect, remains a pressing global health concern. In the United States alone, over 600,000 cases of child maltreatment are reported to the child welfare system annually (U.S. DHHS, 2023). Prior research has largely focused on the consequences of child maltreatment including the adverse effects of maltreatment on youth developmental outcomes (Asgeirsdottir et al., 2011; Shin et al., 2019; Yoon et al., 2018), yet its influence extends beyond individual development and shapes how adolescents interact with their environments (Yoon et al., 2024a, 2024b). As youth transition into adolescence, their engagement with out-of-home environments—such as activity spaces—becomes increasingly important for their mental and physical well-being (White et al., 2021). Thus, identifying how maltreatment histories shape adolescents’ exposure to adversity in their *activity spaces* —the locations that one regularly accesses as part of their daily routines (Browning & Soller, 2014)— is essential to youth well-being. Drawing from social-ecological theory (Bronfenbrenner, 1994; Sallis et al., 2015), individuals are nested within greater community systems (e.g., neighborhoods) that influence their behavior. Therefore, how youth with maltreatment experiences engage in their daily activity spaces may be further shaped by the level of residential neighborhood disadvantage they face. A better understanding of how child maltreatment and neighborhood disorder influence youth activity spaces is critical to inform trauma-informed, community-level interventions aimed at mitigating the adverse impact of child maltreatment and adolescents’ everyday adversity exposure. This study examined the individual and collective influence of maltreatment and neighborhood disorder on adolescents’ exposure to physical and social disorder in their activity spaces.

### Child Maltreatment and Youth Activity Spaces

Activity spaces are important to consider when conducting research with adolescents, as youth in this developmental stage are more likely to spend time outside of the home and with people who are not members of their immediate family (Phillips & Shonkoff, 2000). Emerging evidence shows the value of assessing youth exposure to community processes via activity spaces (Browning et al., 2021; Browning & Soller, 2014). The assessment of youth activity spaces allows for the identification of available places and contextual exposures within and around one’s neighborhood (White et al., 2021). The availability of and experiences in these spaces often reflect environmental resources, personal preferences, and individual needs.

To date, little research has examined the association between child maltreatment and exposure to risks within youth activity spaces (Yoon et al., 2024a, 2024b). This is a critical area of inquiry, as adolescents exposed to maltreatment may have access to different activity spaces or spend more time in risky environments compared to their non-exposed peers, which may serve as a contextual mechanism for sustaining some adverse impacts of child maltreatment. General Strain Theory posits that exposure to strains or stressors can produce negative emotions, such as anger or frustration, which may lead individuals to engage in maladaptive behaviors as a coping mechanism to mitigate stress and reduce negative emotions (Agnew, 1992). Drawing from this theoretical framework, youth who experience maltreatment may intentionally spend more time outside the home to avoid abusive interactions with caregivers and the associated negative emotions. This increased time in public spaces may, in turn, heighten their exposure to risky or disordered environments. Moreover, youth who live in neglectful home environments may have less monitoring and oversight over the places they are allowed to go, potentially increasing their exposure to adversity in their community spaces. Finally, youth who have experienced maltreatment are more likely to be placed in out-of-home care, change schools more frequently, run away from home, or be unhoused (Ferguson, 2009; Kim et al., 2009). These circumstances often reduce supervision, which may increase time spent in environments characterized by high physical and social disorder (Barn & Tan, 2015; Freisthler et al., 2009). *Physical disorder* refers to the amount of visible disarray in an area, including graffiti on buildings and walls, empty cans or bottles, broken glass on the street, and abandoned storefronts (Sampson & Raudenbush, 1999). *Social disorder* refers to the presence of people drinking, fighting, or dealing drugs in community spaces (Sampson & Raudenbush, 1999). Exposure to physical and social disorder is a significant concern, as it is linked to negative outcomes in adolescence, including externalizing behaviors (Gold & Nepomnyaschy, 2018), substance use (dos Santos et al., 2023), and internalizing behaviors (Berg, 2025; Dawson et al., 2019), particularly for youth with a history of child maltreatment (Keyes et al., 2012).

Recent research found that youth with a greater number of maltreatment reports made to child protective services were more likely to be exposed to heightened levels of aggression and violence within their daily activity spaces (Yoon et al., 2024a, 2024b). Still, little is known regarding the relationship between child maltreatment and physical and social disorder within youth activity spaces. Further, most studies rely on self-report measures to assess youth’s environmental exposure, which is constrained by individuals’ attention, memory, and subjective recall bias. By integrating real-time data on youth’s physical locations obtained through the global positioning system (GPS) with aggregated community reports of disorder in specific areas, this study provides a comprehensive, objective portrait of individual activity spaces (Browning & Soller, 2014; Nabi et al., 2023). This approach enables a more accurate analysis of how maltreatment may impact youth activity spaces.

### The Role of Neighborhood Disorder

When examining the association between child maltreatment and youth exposure to disorder in their activity spaces, it is important to consider how this association may vary based on the level of neighborhood disorder in the youth’s home setting because the characteristics of the neighborhood environment may shape the way that youth engage in their activity spaces. Neighborhood disorder, one mechanism by which neighborhood disadvantage influences health and behavioral outcomes (Kim, 2010; Ross & Mirowsky, 2001), includes youth’s exposure to both physical and social disorder within their communities. Emerging evidence suggests that neighborhood disorder is linked to youth activity spaces. Indeed, recent work on activity spaces highlights that the amount of time spent in accessible activity spaces is influenced by the neighborhood disorder-related processes and school accessibility (Browning et al., 2021). Specifically, this study found that youth living in communities with greater neighborhood violence spent more time at home whereas the absence of a neighborhood school was associated with more outside-home-neighborhood time (Browning et al., 2021).

Moreover, experiences of maltreatment may interact with the neighborhood environment in shaping youth activity spaces. Past research suggests a positive link between neighborhood disadvantage and child maltreatment. For example, youth living in systematically disadvantaged neighborhoods, or those experiencing higher rates of poverty, greater substance misuse, less resource access, and more disorder (e.g., broken bottles on streets, boarded up buildings) are at a greater risk for child maltreatment (Barboza-Salerno, 2020; Maguire-Jack & Font, 2017) and child welfare involvement (Fong, 2019; Molnar et al., 2016). Notably, previous studies show that positive neighborhood characteristics, such as neighborhood safety and affluence, can buffer the detrimental effects of child maltreatment on youth behavioral health outcomes (Flouri & Sarmadi, 2016; Kliewer et al., 2004; Vanderbilt-Adriance & Shaw, 2008). The presence of supportive adults (e.g., mentors) within areas youth often visit are also linked with better physical and mental health functioning among youth involved in the child welfare system (Taussig & Culhane, 2010). The absence or low levels of neighborhood disorder and more positive community connections may act as contextual protective factors shielding children with maltreatment histories from engaging in disordered activity spaces. Overall, however, adolescents spend significant portions of their waking time, 34%, outside of their home neighborhood, and just 5.7% within their home neighborhood (Browning et al., 2021). They also experience substantial heterogeneity in neighborhood characteristics in the course of their everyday mobility (Browning et al., 2021). Therefore, capturing how child maltreatment experiences and neighborhood disorder interact and shape adolescents’ exposure to physical and social disorder within the locations that they frequent daily (i.e., activity spaces) is an important step forward in understanding adolescent mental and behavioral health risks.

## Current Study

Research has yet to examine how child maltreatment may be associated with youth’s exposure to physical and social disorder within their daily activity spaces while considering the impact and context of neighborhood disorder. It is critical to understand the relationship between child maltreatment and youth activity space disorder, as the potential findings can inform the strategic placement of community resources within disadvantaged communities. In line with the social-ecological model, an individual’s personal experiences, such as child maltreatment, can intersect with broader environmental factors, such as neighborhood disorder, and contribute to heightened risk exposure within youth’s activity spaces. Thus, it was hypothesized that child maltreatment will be associated with higher levels of physical and social disorder in youth activity spaces (Hypothesis 1), and that child maltreatment will have stronger associations with physical and social disorder in activity spaces for adolescents who live in neighborhoods with higher levels of disorder (Hypothesis 2).

## Methods

### Study Design and Data Sources

This study used two datasets: the Adolescent Health and Development in Context (AHDC) and the Statewide Automated Child Welfare Information System (SACWIS). The AHDC study is a longitudinal survey designed to examine youth’s experiences, health, and well-being in the context of their daily spatial and social environments. Conducted in a Midwestern state in the United States, the study involved 1405 youth aged 11 to 17, and one of their caregivers randomly selected using a sampling frame of vendor- and school-based address lists. AHDC participant households closely approximate the target population of households in the study area with respect to household income and racial composition (Browning et al., 2021). The American Association for Public Opinion Research Response Rate 3—the number of completed interviews out of households either known or estimated to be eligible—was 21.3 percent.

Data collection occurred in 2014–2016 during weeklong periods across two waves: Wave 1 and Wave 2. Each wave began with an entrance survey administered to both youth and their caregivers, gathering detailed information on demographics, socioeconomic status, physical and mental health, social support, family dynamics, parenting practices, behavioral outcomes, and geographic routines. Following the entrance survey, participants completed a 7-day, smartphone-based ecological momentary assessment (EMA), during which real-time data were collected on youth activity spaces, contextual characteristics, and exposures to various risk factors (e.g., substance use, aggression), along with continuous GPS data collection. At the end of the week, youth participants completed a space-time diary with the interviewer, which walked participants through five days of GPS data (including Friday through Sunday and the two most recent weekdays) to summarize stationary and travel periods into a continuous diary of locations, presence of social network partners, and activities (Boettner et al., 2019; Browning et al., 2021).

SACWIS is a comprehensive statewide case management system used by public child welfare agencies. It contains detailed records and information on case reports, including types of alleged child abuse and neglect, investigation outcomes, placement histories, and risk assessment. SACWIS also contains demographic information, such as the youth and caregivers’ name, date of birth, gender, address, and languages spoken.

For this study, the AHDC dataset was linked to the SACWIS dataset. The AHDC participants consented to link administrative data to their AHDC data. Probabilistic matching techniques were employed using various identifiers, including the youth’s first name, middle name, last name, sex, race, date of birth, partial (last two digits) Social Security Number (SSN), and caregiver information, such as first name, last name, date of birth, and partial SSN. Link Plus, a software developed by the Centers for Disease Control and Prevention (n.d.), was used for data matching and linking, ensuring a robust and accurate integration of datasets.

### Measures

#### Child maltreatment

Adolescents’ history of child maltreatment was assessed using SACWIS on the cumulative number of maltreatment allegation reports (e.g., physical abuse, sexual abuse, emotional abuse, neglect) made to child protective services (CPS) from birth to age 17. To address skewness in the data, the number of maltreatment allegation reports was recoded into a dichotomous variable: 0 = no report, 1 = one or more reports.

#### Neighborhood disorder

Neighborhood disorder was calculated by averaging the physical and social disorder measured at the Census tract level, aggregated from reports made by caregivers about social climate perceptions in their residential neighborhood and at routine activity locations. Caregivers are asked to list locations they go to regularly for activities, including work, child’s school, grocery stores, relative and friend’s houses, places of worship, and other stores and businesses. Caregivers then answered a series of questions about the physical and social climate at these locations. Specifically, caregivers were asked to report the extent to which they agree that the items on the physical disorder subscale are a problem (8 items: e.g., cigarettes on the street; graffiti on buildings and walls; garbage and litter; junk vehicles or abandoned cars; vacant buildings or deserted houses) and the social disorder subscale (5 items: e.g., prostitutes on the street; adults or teenagers causing trouble; drug dealing; public intoxication). The original response options ranged from 1 = a big problem to 3 = not a problem. Ratings on 9240 locations from 1353 caregivers within the study area are included. These disorder ratings are modeled using a Bayesian “land use-filtering” spatial statistical approach (see Carter et al., 2024 for more details), which adjusts for the time of day and day of the week of typical visits as well as land use type (e.g., commercial, residential, or other). The model produced spatially smoothed point-level predictions at a small spatial scale by incorporating land use-specific estimates of spatial dependency, creating estimates of neighborhood disorder across the entire study area. Neighborhood disorder is the tract-level disorder value for the tract in which the household resides. Disorder ratings were reverse coded from the original survey questions so that higher scores indicated higher levels of neighborhood disorder.

#### Activity space disorder

A youth-level measure of activity space disorder was calculated by combining the average level of disorder within census block groups and adjusting for the amount of time the youth spent in each block group. Specifically, activity space disorder is based on exposures to physical and social disorder at the locations visited during the EMA/GPS week and recorded in the space-time diary. The point-level predictions from the land-use filtering model of disorder were averaged to the Census block group level using the same method as the neighborhood tract aggregation described above. Block groups in the study area have on average 15.50 caregiver location reports (*SD* = 21.18) from an average of 12.62 unique caregivers (*SD* = 16.90) that contribute to the spatially smoothed point-level predictions. Only 32 of 614 block groups in the study area have 0 caregiver reports contributing to the land-use filtering prediction model; youth spent a median of 0 min in these block groups across the week of space-time diary data; the 90^th^ percentile of the youth distribution is just 2 min spent in these block groups. Non-home stationary (non-travel) locations from the space-time diary were geocoded to the Census block group and linked to block group aggregated measures. The average disorder level experienced by the youth across the week is then weighted for the amount of time spent in each block group. Therefore, block groups in which youth spend more time contribute more heavily to their weekly average level of activity space disorder exposure. Higher scores indicated higher levels of disorder within youth activity spaces.

#### Covariates

Based on the literature (e.g., Mennis & Mason, 2012), models controlled for the following constructs that are associated with youth activity space characteristics: 1) youth gender (0 = girls, 1 = boys), 2) youth age, 3) youth race (0 = non-Black, 1= Black), 4) proportion of time youth spent at home, 5) caregiver education, and 6) household income. Time spent at home was measured as the proportion of waking time spent at home. This variable ranged from 0 to 1, representing the relative amount of awake time spent at home. Caregiver education was measured using caregiver self-reports of their highest level of educational attainment and included five categories: 1 = less than high school, 2 = graduated high school, 3 = college, 4 = bachelor’s, 5 = graduate school. Household income was measured using four categories based on annual income in the last year: 1 = $30,000 or less, 2 = $30,001 - $60,000, 3 = $60,001 - $150,000, and 4 = $150,000 or more. Both caregiver education and household incomes were treated as continuous for analytical interpretation.

### Data Analytic Plan

To explore the effect of maltreatment experiences and residential neighborhood-level disorder on exposure to physical and social disorder in youth activity spaces, multilevel modeling was conducted. The dataset in this study has a multilevel structure where youth are nested within residential neighborhoods. The nested data structure violates the assumption of independence and can increase Type I error (Barcikowski, 1981; Curran, 2003; Hox, 1998; Snijders & Bosker, 2011). To address the issue of data dependency (e.g., intraclass correlation; ICC), multilevel modeling was implemented which allows for the examination of the individual- and group-level predictors, differences between groups, and cross-level interactions (i.e., interactions between an individual-level variable and a cluster-level variable).

The following models were examined by adding covariates sequentially: 1) baseline model and ICC, 2) model with individual-level covariates, 3) model with individual- and neighborhood-level covariates, and 4) model with cross-level interaction. The most complex model, which included individual-level covariates, neighborhood-level covariates, and cross-level interaction, is represented as follows:

$$\begin{array}{c}{{Activity\; Space\; Disorder}}_{{ij}}={\gamma }_{00}+{\gamma }_{01}{{Neighbordood\; Disorder}}_{j}+{\gamma }_{10}{{Gender}}_{{ij}}+{\gamma }_{20}{{Age}}_{{ij}}+{\gamma }_{30}{{Black}}_{{ij}}\\ +{\gamma }_{40}{{Caregiver\; edu}}_{{ij}}+{\gamma }_{50}{{Household\; income}}_{{ij}}+{\gamma }_{60}{{Time\; spent\; at\; home}}_{{ij}}+{\gamma }_{70}{{Maltreatment}}_{{ij}}\\ +{\gamma }_{71}{{Maltreatment}}_{{ij}}{{Neighborhood\; Disorder}}_{j}+({u}_{0j}+{u}_{7j}{{Maltreatment}}_{{ij}}+{r}_{{ij}})\end{array}$$

Here, *i* and *j* denote an individual and index of his/her neighborhood, respectively. For instance, ${u}_{0j}$ represents the deviation of neighborhood *j* from the grand mean disorder. The $r$ represents individual-level error/variability, capturing the deviation of an individual’s observed youth activity space disorder from the group’s (e.g., neighborhood) predicted value, not explained by the predictors.

${\gamma }_{10}$ – ${\gamma }_{70}$ are the effects of individual-level predictors on the outcome. The ${\gamma }_{01}$ is the main effect of neighborhood-level disorder. Lastly, ${\gamma }_{71}$ is the interaction coefficient, which allows for the exploration of whether the effect of maltreatment on the outcome varies by the level of neighborhood disorder. The *γ*s denote the fixed effects while the *u*s denote random effects. The fixed effect indicates the average association between the predictor (i.e., maltreatment) and youths’ exposure to disorder in their activity spaces, while the random effect captures how this association may differ from one neighborhood to another.

Individual-level continuous covariates were group-mean centered, and the neighborhood-level covariate (i.e., the neighborhood disorder measure) was grand-mean centered. All analyses were conducted using *Mplus* Version 8.0. Data were missing for 162 cases on one or more exogenous variables and for 27 cases on endogenous variables. Missing data were addressed using listwise deletion, as the model included a random slope and full information maximum likelihood (FIML) is not readily available for such models in Mplus. Prior research also supports listwise deletion as a preferable alternative to multiple imputation when random slopes are included in the model (Grund et al., 2016). The final analytic sample consisted of 1215 youth.

## Results

Table 1 presents the descriptive statistics and mean values for key variables across the two groups (with and without maltreatment history). Youth in the group with a maltreatment history (youth with at least one maltreatment allegation reported to child protective services) visited activity spaces with significantly higher levels of disorder compared to youth in the group without maltreatment history (*t* = −8.16, *p* < 0.001). The group with a maltreatment history had a higher proportion of girls. Racial/ethnic composition also varied, as over half of the non-maltreatment group identified as White (50.7%), whereas the majority of the group with maltreatment history identified as Black (54.9%). The distributions of caregiver education and household income varied between the groups as well. The group with a maltreatment history had a higher proportion of caregivers with less than a high school education and families with incomes below $30,000. There was no statistically significant difference in the proportion of waking time spent at home (*t* = −0.976, *p* = 0.165). Lastly, the neighborhoods where youth in the maltreatment history group resided showed significantly higher levels of disorder compared to those where youth in the non-maltreatment group lived (*t* = −8.00, *p* < 0.001). Correlations among the study variables are shown in Table 2.

**Table 1:** Descriptive Statistics and Sample Characteristics

	Without maltreatment history (*N* = 978)	With maltreatment history (*N* = 237)	*t*	*p*
	%/ Mean (SD)	%/ Mean (SD)		
*Individual-level*				
Disorder of activity space	1.19 (0.15)	1.28 (0.15)	−8.16	<0.001
Gender (% of boys)	48.2%	42.6%		
Age	14.38 (1.83)	13.74 (1.91)	4.79	<0.001
Race ethnicity (%)				
White	50.7%	28.3%		
Black	33.6%	54.9%		
Any Hispanic	5.8%	5.1%		
Asian	1.8%	0.0%		
Multi-racial	7.9%	11.4%		
Native American	0.1%	0.4%		
Caregiver education (%)				
Less than high school	3.7%	9.7%		
Graduated high school	14.4%	18.6%		
College	31.6%	54.9%		
Bachelor	27.8%	11.8%		
Graduate school	22.5%	5.1%		
Household income (%)				
$30,000 or less	29.2%	64.6%		
$30,001–$60,000	23.7%	25.3%		
$60,001–$150,000	32.9%	8.0%		
$150,000 or more	14.1%	2.1%		
Time spent at home	0.60 (0.20)	0.61 (0.21)	−0.976	0.165
*Neighborhood-level*				
Disorder	1.22 (0.19)	1.33 (0.18)	−8.00	<0.001

N = 1215; SD: standard deviation

**Table 2:** Correlations among Key Study Variables

	1	2	3	4	5	6	7	8	9
1. Activity space disorder	—								
2. Maltreatment	0.25***	—							
3. Gender (boys)	−0.01	−0.04**	—						
4. Age (years)	−0.08**	−0.13***	−0.07*	—					
5. Race (Black)	0.52***	0.17***	−0.01	−0.06*	—				
6. Caregiver education	−0.48***	−0.25***	0.000	0.06*	−0.34***	—			
7. Household income	−0.56***	−0.33***	−0.001	0.09*	−0.43***	0.59***	—		
8. Time spent at home	0.07*	0.03	0.07*	−0.07*	0.01	−0.09**	−0.11***	—	
9. Neighborhood disorder	0.76***	0.24***	0.02	−0.10	0.53***	−0.52***	−0.59***	0.06	—
10. Time spent outside of home block	−0.001	−0.03	−0.08*	0.05	0.04	0.05	0.06	−0.88**	0.02

N = 1215; *p < 0.05. **p < 0.01. ***p <0.001

Table 3 presents the results of the multilevel modeling analysis examining the effects of individual- and neighborhood-level covariates on disorder levels in youth activity spaces. Model 1 (baseline unconditional model) provided information on the overall level of activity space disorder where youth visited and where the variances come from. The ICC was 0.504, indicating that more than half of the variation in youth’s individual-level activity space was attributable to neighborhood characteristics, and further analyses were needed.

**Table 3:** Multilevel Analysis Models of Predictors of Activity Space Disorder

	Model 1		Model 2		Model 3		Model 4	
Fixed effect	Coef.	SE	Coef.	SE	Coef.	SE	Coef.	SE
Intercept (${\gamma }_{00}$)	1.221^***^	0.009	1.198^***^	0.010	1.202^***^	0.007	1.202^***^	0.007
Individual-level								
Boys (${\gamma }_{10}$)			−0.008	0.007	−0.008	0.006	−0.008	0.006
Age (${\gamma }_{20}$)			−0.002	0.002	−0.002	0.002	−0.002	0.002
Black (${\gamma }_{30}$)			0.062^***^	0.011	0.049^***^	0.010	0.049^***^	0.010
Caregiver education (${\gamma }_{40}$)			−0.002	0.006	−0.002	0.006	−0.002	0.006
Household income (${\gamma }_{50}$)			−0.014	0.005	−0.014^*^	0.005	−0.014^*^	0.005
Time spent at home (${\gamma }_{60}$)			0.014	0.018	0.013	0.018	0.013	0.018
Maltreatment (${\gamma }_{70}$)			0.019^*^	0.009	0.013	0.009	0.013	0.009
Neighborhood-level								
Disorder (*γ*_01_)					0.488^***^	0.031	0.496^***^	0.031
Cross-level interaction								
Maltreatment × Disorder (${\gamma }_{71}$)							−0.059	0.047
Random effect								
Individual-level (${r}_{{ij}}$)	0.011^***a^	0.001	0.011^***^	0.001	0.011^***^	0.001	0.011^***^	0.001
Intercept (${u}_{0j}$)	0.012^***b^	0.001	0.009^***^	0.001	0.001^**^	0.000	0.001^**^	0.000
Maltreatment (${u}_{7j}$)			0.001	0.001	0.001	0.001	0.001	0.001

N = 1215; Model 1: baseline model, Model 2: model with individual-level covariates, Model 3: model with individual- and neighborhood-level covariates, Model 4: model with cross-level interaction; a = 0.0116, b = 0.0118. SE = standard errors

* *p* < 0.05, ** *p* < 0.01, *** *p* < 0.001

Model 2 examined the effects of child maltreatment on disorder levels in youth activity spaces, while controlling for individual-level covariates, such as youth gender, race, caregiver education, household income, and youth-reported time spent at home. There was a significant positive association between child maltreatment and activity space disorder (${\gamma }_{70}$ = 0.019). Youth who had one or more child maltreatment reports were more likely to visit places with higher levels of disorder during their daily routine. There were other individual-level covariates that were also significantly associated with activity space disorder. Black youth were more likely to visit disordered places than non-Black youth (${\gamma }_{30}$ = 0.062). Likewise, household income was negatively associated with disorder in youth’s activity space. That is, youth in more affluent households were more likely to visit places with lower levels of disorder (${\gamma }_{50}$ = −0.014).

Model 3 examined the effect of neighborhood-level disorder on activity space disorder while controlling for the individual-level covariates. The positive and significant coefficient of neighborhood-level disorder indicated that youth who lived in neighborhoods with higher levels of disorder were more likely to visit places with higher levels of disorder compared to those living in neighborhoods with lower levels of neighborhood disorder (${\gamma }_{01}$ = 0.488).

Lastly, Model 4 examined the cross-level interaction by examining the effect of neighborhood-level disorder on the maltreatment effect. The interaction was not statistically significant, indicating that neighborhood-level disorder did not modify the positive association between child maltreatment and youth’s exposure to disorder in activity spaces. Both child maltreatment (${\gamma }_{70}$ = 0.018) and neighborhood disorder (${\gamma }_{01}$ = 0.496) remained significant predictors of activity space disorder in Model 4.

## Discussion

A deeper understanding of how child maltreatment shapes adolescents’ exposure to risky environments in their daily lives is crucial for promoting positive youth development and well-being. To date, limited research has examined how maltreatment experiences influence adolescents’ daily experiences beyond their residential neighborhoods. Investigating the places and locations that youth visit during their daily activities (i.e., youth activity spaces) is particularly important given the increased mobility and spatial exposure that occurs during adolescence (Browning et al., 2021). An innovative approach that linked child welfare administrative records with GPS-based youth activity space data was used to evaluate the association among child maltreatment, neighborhood disorder, and adolescents’ exposure to physical and social disorder within their activity spaces with great precision and robustness.

Findings indicate that adolescents with a higher number of maltreatment reports (child maltreatment allegations made to child protective services) are more likely to visit places with higher levels of physical and social disorder during their daily routine. Prior research suggests that victims of maltreatment are more prone to risk-taking propensity due to altered brain development associated with maltreatment, including reduced reward sensitivity and diminished impulse control (Mueller et al., 2010; Whittle et al., 2013). Such impaired cognitive control (e.g., disinhibition) and a diminished ability to accurately appraise risk (Bornovalova et al., 2008; Smith et al., 2004) may place youth with maltreatment histories at greater risk for exposure to risky and unsupervised activity spaces, such as locations with higher levels of social (e.g., people drunk or taking drugs on the streets, drug dealing) and physical (e.g., vacant or abandoned housing) disorder (Ross & Jang, 2000; Ross & Mirowsky, 1999). Importantly, the relationship between child maltreatment exposure and heightened activity space disorder may partially explain why maltreated youth may be more likely to engage in their own risk-taking behavior. Youth who witness greater levels of disorder within their daily activity spaces may normalize these behaviors or view them as less risky or harmful, resulting in personal decisions to use substances or participate in delinquent activities. Though the causal relationship between these variables is outside the scope of this study, future work could explore the potential mediating effects of activity space disorder on the relationship between child maltreatment and youth outcomes (e.g., substance use).

Additionally, youth with a history of maltreatment may choose to spend more time outside the home to protect themselves from potential harm occurring at home. Supervisory neglect or emotional abuse may also increase their risk of associating with deviant peers, which could further expose them to unsafe and unstructured environments (Yoon et al., 2019). The finding that maltreatment history explains variations in youth activity disorder above and beyond the impact of residential neighborhood disorder highlights that youth activity spaces extend beyond the confines of their home neighborhoods and that youth exercise their agency in selecting their environments. Results demonstrate the importance of a comprehensive examination of youth’s social and spatial exposure, beyond the home neighborhood environment. The findings also highlight the need for targeted support and interventions for adolescents with a history of child maltreatment to reduce their future exposure to high-risk environments and foster safer developmental pathways.

Findings also reveal a positive association between neighborhood disorder and the level of disorder within youth activity spaces. This interconnection may be explained by a spillover effect (Nilsson et al., 2017), in which disordered conditions in one setting (e.g., a residential neighborhood) extend into other settings (e.g., activity spaces). Adolescents with limited mobility (e.g., lack of access to a car) may regularly visit and spend time in locations proximal to their home neighborhoods, leading youth from highly disadvantaged and disordered areas to experience similar levels of disorder in their activity spaces (e.g., recreational areas). Youth from disordered neighborhoods may also have lower physiological responses to visiting places with high disorder. Adolescents accustomed to neighborhood disorder (e.g., substance use on streets, and abandoned housing) may become desensitized to such environments and feel more comfortable and tolerant of spending time in similarly disordered spaces (Schwab-Stone et al., 1995). To promote youth safety and well-being, practitioners and stakeholders should consider implementing measures to address neighborhood-level disorder. Examples include investing in community green spaces in under-resourced areas, increasing access to small business loans for disadvantaged communities, or supporting large-scale efforts to reduce substance use and increase treatment access. Such strategies could result in less neighborhood disorder and reduced adversity exposure among adolescent residents. It is worth noting that youth’s perceptions of disorder within their activity spaces and their reasons for spending time in their activity spaces were not assessed (e.g., a job, sports practice, hanging out with friends). Additional work is needed to assess how youth perceive disorder within their activity spaces and clarify how youth spend their time in their activity spaces as these potential differences could attenuate poor outcomes following exposure to maltreatment.

Interestingly, neighborhood disorder did not moderate the relationship between maltreatment history and exposure to activity space disorder. In other words, adolescents with a history of child maltreatment remained at greater risk for exposure to disordered activity spaces, regardless of the level of disorder in their home neighborhoods. It is plausible that, regardless of neighborhood context, caregivers who perpetrate maltreatment may spend more time in spaces with greater levels of disorder (e.g., substance use) compared to caregivers without maltreatment histories. This could partially explain why the link between child maltreatment and youth’s activity space disorder was not influenced by the level of neighborhood disorder. Alternatively, the absence of protective effects from low-disorder neighborhoods may support the idea that maltreatment-related alterations in brain development and impaired cognitive control may drive risk-taking behaviors during adolescence (Teicher et al., 2016). In addition, from the perspective of developmental psychopathology (Sroufe & Rutter, 1984), the timing of experiences and the changes in contexts can significantly shape children’s developmental outcomes (Yoon et al., 2024a, 2024b). Children who experience maltreatment may experience increased instability in their living arrangements (Herrenkohl et al., 2003). The protective effects of contextual factors, such as residing in a low-disorder neighborhood, may take time to become evident or may only emerge when there is a certain degree of stability within children’s neighborhood environments. Future studies with longitudinal data are warranted to examine these speculations and further explore other protective contextual factors (e.g., community cohesion, safe community spaces).

Additionally, the current study observed consistent links between racial status and youth activity space disorder as well as between household income and youth activity space disorder, which suggests that the structural inequalities created by racism and financial strains put youth at further disadvantages. Structural racism and classism have maintained discriminatory housing practices (e.g., red lining) that place Black families and lower-income families in communities with limited resource access (Littleton et al., 2024). These social conditions maintain economic inequality (e.g., poverty), contributing to concentrated disadvantage and subsequent neighborhood disorder and activity space disorder that place youth at risk for additional adversity exposure. Notably, Black youth and youth from lower-income families are overrepresented in the child welfare system (Cénat et al., 2021) and were overrepresented in the current study’s subgroup of youth with a history of child welfare involvement. Future studies should attempt to corroborate child welfare reports of maltreatment with self-reported measures to gain a more accurate picture of youth’s maltreatment exposure and explore potential differences in youth’s outcomes based on the source of reported maltreatment. Finally, it remains a critical issue to systematically address the multiple layers of disadvantage created by structural inequality that maintain resource-deprived communities, resulting in an overreporting of racial minority families and lower-income families to child welfare.

### Limitations and Future Directions

The study has several limitations. First, the generalizability of the findings is limited, as participants were recruited from a single city in a Midwest state. Second, child welfare administration records, specifically the number of maltreatment reports made to child protective services, were used to assess adolescents’ maltreatment experiences. This approach does not fully capture the severity or frequency of maltreatment exposure or include youth who may have experienced maltreatment but were not reported to child welfare. Future research should consider applying a longitudinal design to explore the mechanisms through which maltreatment shapes adolescents’ experiences in activity spaces. Third, the physical and social disorder measures are based on caregivers’ self-reports of perceived conditions rather than a direct observation, street audit approach, or adolescents’ own perceptions of physical and social disorder. Caregivers’ reports of these conditions may not include all aspects of neighborhood and activity space disorder relevant for adolescent outcomes (for example, see Mooney et al., 2014). Future work should assess youth’s perceptions of their activity spaces and their reasons for going to their activity spaces (e.g., job, socializing) as these variables may influence their exposure to activity space disorder. Additionally, future studies explore protective factors that can facilitate resilience within youth’s activity spaces (e.g., mentors, green space, structured activities, community service) and examine the potential moderating effects of these variables between child maltreatment and adolescents’ developmental outcomes. Finally, the measures of activity space exposures are only based on the 5-day space-time diary constructed from summarized GPS data, which does not fully capture the extent of adolescent activity exposures across a longer time period.

### Implications

Study findings offer significant implications for future research, practice, and policy. From a research perspective, this study highlights the value of linking administrative and GPS-based data to study the everyday experiences of adolescents with a history of child welfare involvement. This data linking method could be applied to other national datasets to investigate important research questions, for example, whether GPS-based data more accurately predict youth outcomes than census tract data. Findings from the study highlight the need for strengthening maltreatment prevention efforts and implementing targeted, accessible interventions for adolescents who have experienced child maltreatment. Providers working with youth exposed to maltreatment and their caregivers should communicate risks for future adversity exposure in youth’s daily activity spaces and discuss ways to bolster protective interactions in their activity spaces. For example, providers can support youth in identifying places in their community that could support their well-being. Regarding policy implications, the findings call for increased investment and expansion of public programs and policies that address community-level disorder and disadvantages that can negatively influence adolescents’ daily spatial and environmental exposures. Indeed, addressing both individual and environmental risk factors through practice and policy is essential to effectively support positive developmental pathways for vulnerable youth with a history of child maltreatment.

## Conclusion

Youth who experience maltreatment are at risk for additional adversity exposure both at home and in their larger communities. This study examined how child maltreatment is associated with adolescents’ exposure to physical and social disorder in the places they regularly visit (i.e., activity spaces) and the potential moderating effect of neighborhood disorder on this association. Results revealed that youth who had one or more maltreatment reports were more likely to visit places with higher levels of disorder during their daily routine. Similarly, youth who lived in neighborhoods with higher levels of disorder were more likely to visit places with high disorder compared to those living in neighborhoods with lower neighborhood disorder. Findings highlight the critical need for enhanced maltreatment prevention efforts that target community spaces and aim to increase positive community supports for adolescents (e.g., parks, community centers, libraries), especially those with maltreatment histories. Additionally, results echo other calls to action to increase investments in and the expansion of public programs and policies that address community-level disorder and disadvantage that could reduce adolescents’ future exposure to adversity within their daily activity spaces.
